# Hydroquinones Including Tetrachlorohydroquinone Inhibit Candida albicans Biofilm Formation by Repressing Hyphae-Related Genes

**DOI:** 10.1128/spectrum.02536-22

**Published:** 2022-10-03

**Authors:** Yong-Guy Kim, Jin-Hyung Lee, Sunyoung Park, Sagar Kiran Khadke, Jae-Jin Shim, Jintae Lee

**Affiliations:** a School of Chemical Engineering, Yeungnam University, Gyeongsan, Republic of Korea; University of Debrecen

**Keywords:** biofilm, *C. albicans*, hydroquinone, hyphal inhibition, tetrachlorohydroquinone

## Abstract

Candida albicans is an opportunistic pathogenic fungus responsible for candidiasis. The pathogen readily forms antifungal agent-resistant biofilms on implanted medical devices or human tissue. Morphologic transition from yeast to filamentous cells and subsequent biofilm formation is a key virulence factor and a prerequisite for biofilm development by C. albicans. We investigated the antibiofilm and antifungal activities of 18 hydroquinones against fluconazole-resistant C. albicans. Tetrachlorohydroquinone (TCHQ) at subinhibitory concentrations (2 to 10 μg/mL) significantly inhibited C. albicans biofilm formation with an MIC of 50 μg/mL, whereas the backbone hydroquinone did not (MIC > 400 μg/mL), and it markedly inhibited cell aggregation and hyphal formation. Transcriptomic analyses showed that TCHQ downregulated the expressions of several hyphae-forming and biofilm-related genes (*ALS3*, *ECE1*, *HWP1, RBT5,* and *UME6*) but upregulated hyphae- and biofilm-inhibitory genes (*IFD6* and *YWP1*). Furthermore, it prevented C. albicans biofilm development on porcine skin and at concentrations of 20 to 50 μg/mL was nontoxic to the nematode Caenorhabditis elegans and did not adversely affect Brassica rapa seed germination and growth. This study indicates that hydroquinones, particularly TCHQ, diminish the virulence, biofilm formation, and animal tissue adhesion of C. albicans, which suggests hydroquinones should be considered potential candidate antifungal agents against drug-resistant C. albicans strains.

**IMPORTANCE** Persistence in chronic infections by Candida albicans is due to its ability of biofilm formation that endures conventional antifungals and host immune systems. Hence, the inhibition of biofilm formation and virulence characteristics is another mean of addressing infections. This study is a distinctive one since 18 hydroquinone analogues were screened and TCHQ efficiently inhibited the biofilm formation by C. albicans with significantly changed expressional profile of hyphae-forming and biofilm-related genes. The antibiofilm efficacy was confirmed using a porcine skin model and chemical toxicity was investigated using plant seed germination and nematode models. Our findings reveal that TCHQ can efficiently control the C. albicans biofilms and virulence characteristics.

## INTRODUCTION

Candida albicans is found on skin and in the gastrointestinal tract and normally behaves as a commensal yeast, but can become pathogenic, cause candidiasis, and affect the mouth, genitals, and skin. C. albicans colonizes host tissues and implanted medical devices, such as stents, implants, and catheters (1), and can reversibly switch between yeast and filamentous forms (hyphae), the latter of which plays an important pathogenic role by invading epithelial cells and causing tissue damage (2). Furthermore, this morphogenetic conversion plays a pivotal role in C. albicans biofilm development (1) while the molecular basis of which has been partially unveiled (3).

Biofilm formation results from the encasement of metabolically inactive cells by exopolymeric substances and hinders the diffusion of antifungal agents into cells. Furthermore, it has been widely reported that sessile *Candida* cells are more resistant to antimicrobial agents (3, 4). *Candida* species also include various virulence factors such as membrane and cell wall barriers, dimorphism, biofilm formation, a signal transduction pathway, hydrolytic enzymes (e.g., proteases, lipases, haemolysins), and toxin production (5). Hence, inhibitions of biofilm and hyphal formation are considered alternative means of controlling *Candida* virulence (6). Unlike antifungal agents that target planktonic cell growth, nontoxic agents with antibiofilm and antihyphal activities that suppress virulence traits and pathogenesis without killing microbes are required, as these strategies diminish evolutionary pressure toward the development of drug resistance (7, 8). Therefore, this study was designed to identify nontoxic antibiofilm hydroquinones exhibiting antifungal activity against drug-resistant C. albicans and to investigate the mechanisms responsible.

Diverse natural and synthetic compounds have been reported to inhibit biofilm formation by C. albicans, and several anthraquinones (e.g., alizarin, chrysazin, and purpurin) reportedly inhibit biofilm and hyphal formation by C. albicans by repressing several hyphae-specific and biofilm-related genes (*ALS3*, *ECE1*, *ECE2*, and *RBT1*) (9). However, the structural backbones of these molecules (hydroquinone and anthraquinone) have no or little effect on C. albicans (9). Hence, the current study was undertaken to investigate various hydroquinone derivatives for their antifungal and antibiofilm activities.

In this study, we screened 17 hydroquinone (HQ) derivatives and HQ for antibiofilm activity against an antifungal-resistant C. albicans strain. The effects of the most active compound tetrachlorohydroquinone (TCHQ) and HQ on planktonic growth and hyphal inhibition were further investigated. C. albicans cell morphology and phenotypic switching were observed by live imaging microscopy, confocal laser scanning microscopy (CLSM), and scanning electron microscopy (SEM). In addition, hyphal protrusion and cell aggregation assays were performed to confirm inhibitory effects on hyphal formation. Quantitative real-time reverse transcription-PCR (qRT-PCR) was used to investigate the molecular basis of their activities. In addition, toxicological studies were conducted using a plant seed germination and nematode model to evaluate ecological effects, and a porcine skin *ex-vivo* model was used to confirm antibiofilm effects.

## RESULTS

### Effects of hydroquinone (HQ) and its derivatives on C. albicans biofilm formation and cell growth.

The antibiofilm activities of HQ and the 17 HQ derivatives at 10 or 50 μg/mL were initially examined against C. albicans DAY185 (a fluconazole-resistant strain) on 96-well polystyrene plates. The 18 HQs were found to have widely different antibiofilm activities (Table 1). Three derivatives, namely, 2,5-dibromohydroquinone, TCHQ, and tetrafluorohydroquinone exhibited significant antibiofilm activity while the other 15 HQs did not. At a concentration of 50 μg/mL, 2,5-dibromohydroquinone and TCHQ both inhibited C. albicans biofilm formation by > 97%, but only TCHQ inhibited biofilm formation by > 97% at 10 μg/mL.

**TABLE 1:** Antibiofilm and antibacterial activities of the 18 hydroquinones against C. albicans^*a*^

			Biofilm (%)		Growth (%)	
Hydroquinones	Structures	MIC μg/mL	10	50	10	50
2,5-Bis(1,1,3,3-tetramethylbutyl) hydroquinone	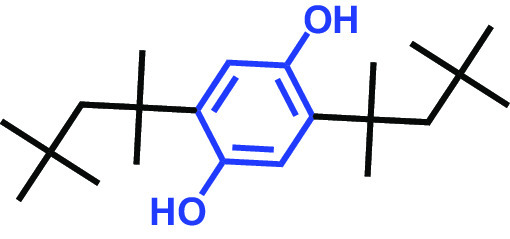	>400	97	98	98	99
Chlorohydroquinone	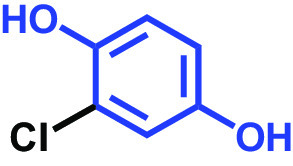	>400	99	91	96	89
2,5-Dibromohydroquinone	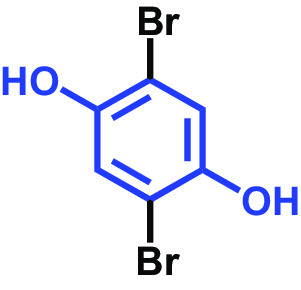	200	100	3	96	30
2,3-Dicyanohydroquinone	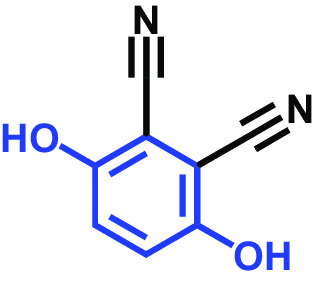	>400	99	99	99	98
2,3-Dimethylhydroquinone	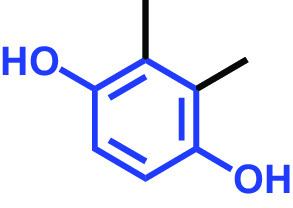	>400	98	99	99	97
2,6-Dimethylhydroquinone	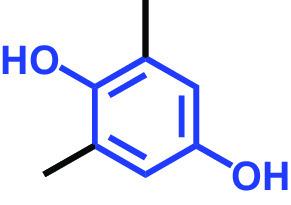	>400	97	94	98	97
2,5-Di-tert-butylhydroquinone	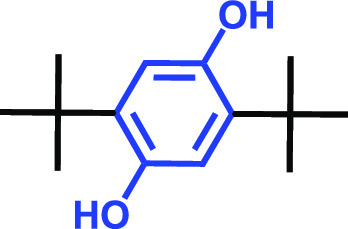	>400	100	90	96	97
Hydroquinone (HQ)	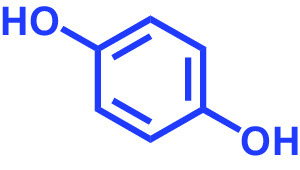	>400	96	96	98	99
Hydroquinone monobenzyl ether	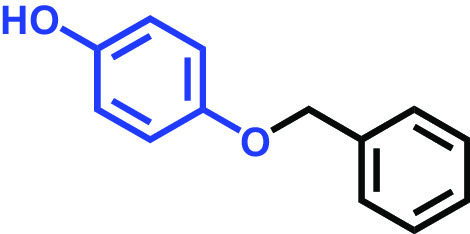	>400	94	91	95	87
Hydroquinone-o,o′-diacetic acid	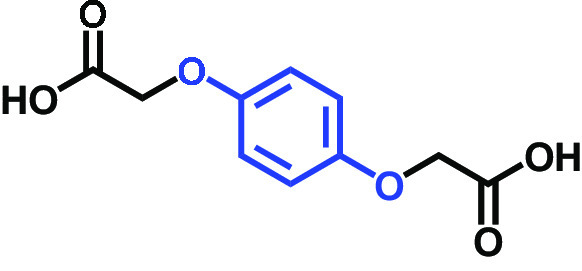	>400	100	100	98	100
Methylhydroquinone	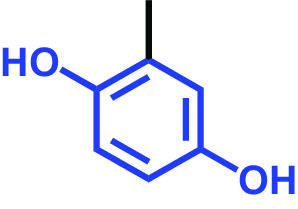	>400	96	98	97	100
2-Methoxyhydroquinone	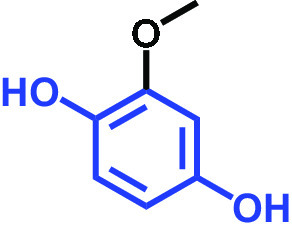	>400	97	96	99	99
4-Methoxyphenol	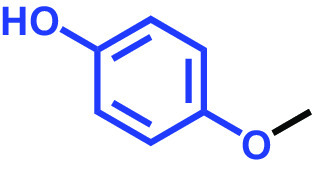	>400	97	96	101	98
tert-Butylhydroquinone	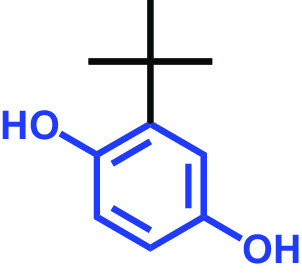	>400	95	90	98	98
Tetrachlorohydroquinone (TCHQ)	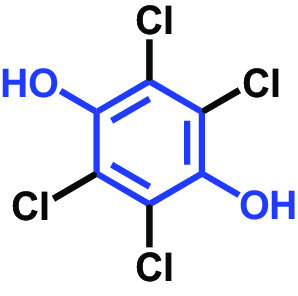	50	3	1	72	10
Tetrafluorohydroquinone	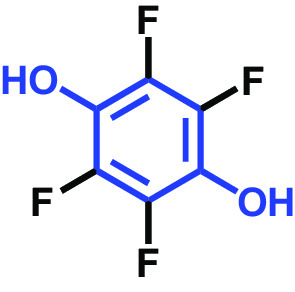	>400	98	69	97	28
4-((Tetrahydro-2H-pyran-2-yl)oxy)phenol	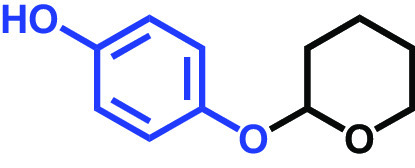	>400	93	96	100	98
Trimethylhydroquinone	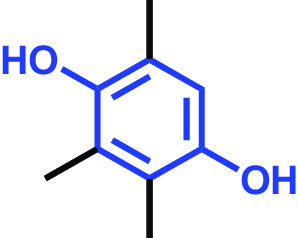	>400	96	100	99	98

a MICs, biofilm formation, and planktonic cell growth of C. albicans DAY185 were determined after incubation for 24 h in 96-well plates.

**FIG 1:**
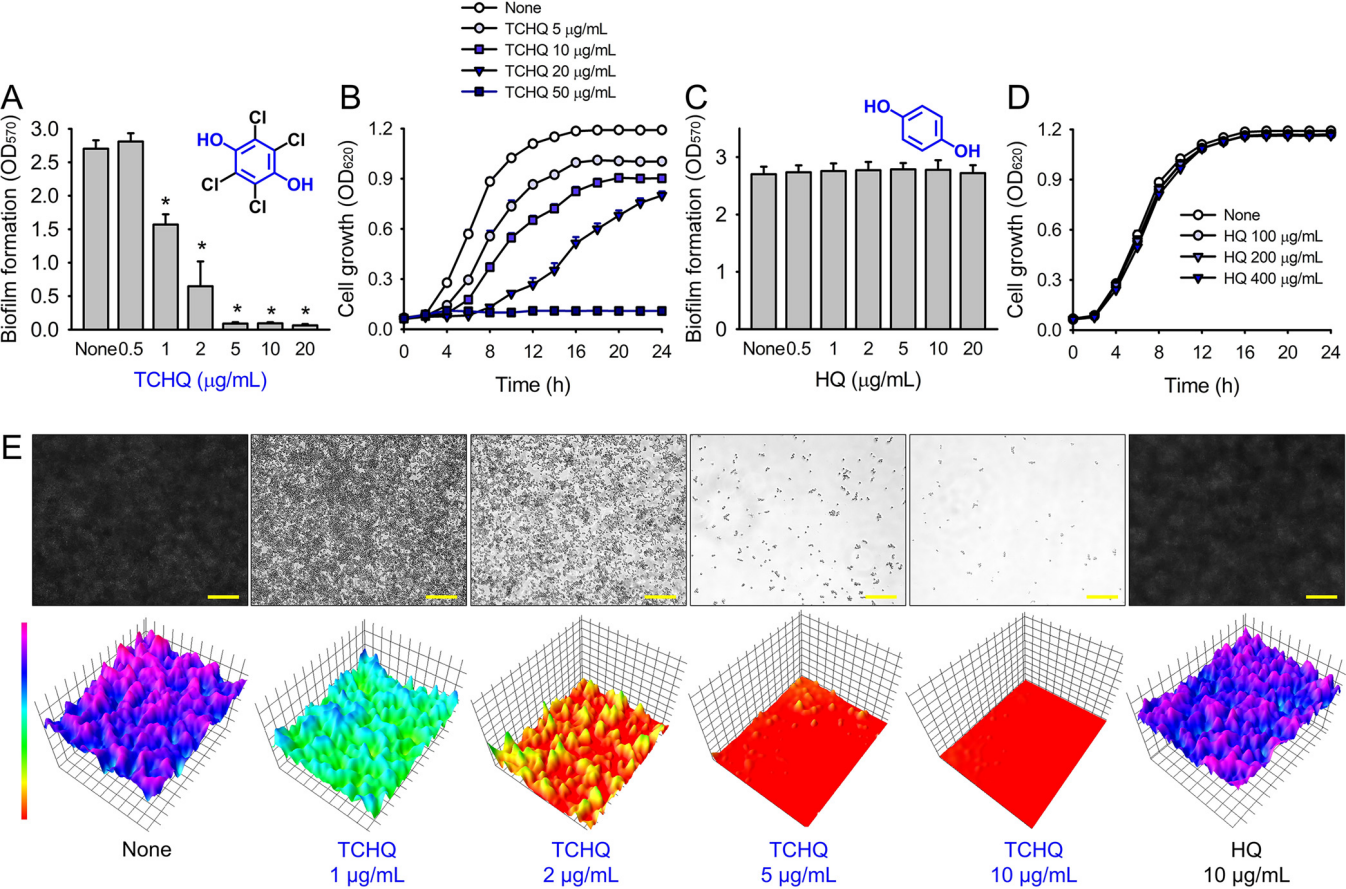
Inhibition of C. albicans biofilm formation by hydroquinones. The antibiofilm activities of TCHQ and HQ on C. albicans DAY185 (A and C) and planktonic cell growth (B and D) were investigated in 96-well polystyrene plates after culture for 24 h. Constructed color-coded 2D and 3D images of C. albicans biofilms after culture with TCHQ or HQ (E). Yellow scale bars indicate 100 μm. Error bars represent standard deviations. *, *P* < 0.05 versus nontreated controls; None, nontreated controls.

The MICs of the 18 HQs were also measured. Most had MICs of > 400 μg/mL, but 2,5-dibromohydroquinone and TCHQ had MICs of 200 and 50 μg/mL, respectively. A more detailed biofilm study showed that TCHQ at lower doses significantly inhibited C. albicans DAY185 biofilm formation (Fig. 1A). For example, at 2 and 5 μg/mL, TCHQ inhibited C. albicans biofilm formation by 76 and 96%, respectively, whereas HQ did not show any activity at concentrations ≤ 100 μg/mL (Fig. 1A to C). Notably, TCHQ at 5 μg/mL (10% of its MIC) markedly inhibited biofilm formation. Furthermore, planktonic cell growth curves confirmed that the MIC of TCHQ was 50 μg/mL and at concentrations between 5 and 50 μg/mL it delayed planktonic cell growth (Fig. 1B), whereas HQ at concentrations of up to 400 μg/mL had no effect (Fig. 1D). Due to its potent antibiofilm and antifungal activities, TCHQ was selected for further assays and HQ was included for comparison purposes. The antibiofilm activity of TCHQ was also tested against a fluconazole-sensitive C. albicans ATCC 10231 strain, and as was expected, TCHQ dose-dependently inhibited its biofilm formation. HQ had no effect (see Fig. S1 in the supplemental material).

### Microscopic assessments of C. albicans biofilm inhibition by TCHQ.

**FIG 2:**
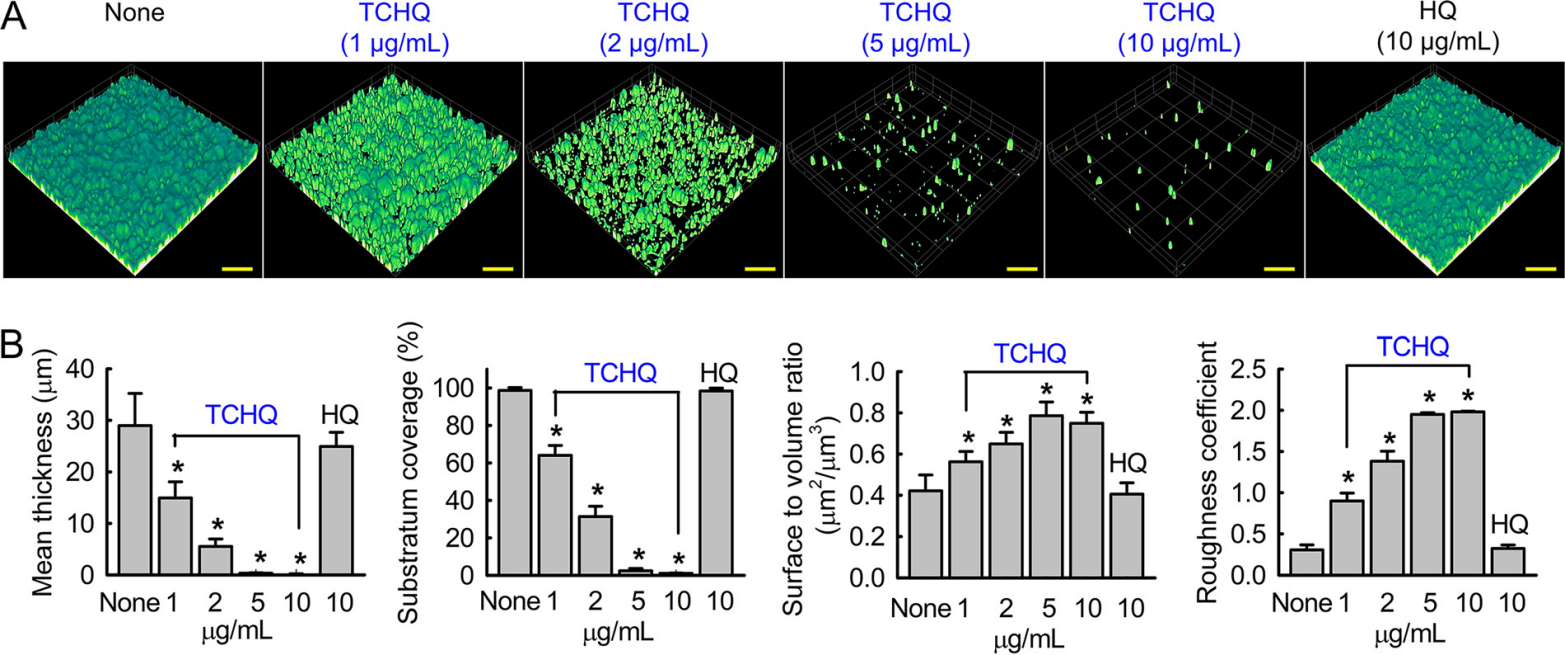
CLSM images of C. albicans biofilm inhibition by TCHQ and HQ (A) and the results of COMSTAT analysis of CLSM images (B). Yellow scale bars represent 100 μm. *, *P* < 0.05 versus nontreated controls (None).

Bright-field microscopy and confocal laser scanning microscopy (CLSM) were used to observe biofilm inhibition by TCHQ. Two- and three-dimensional LUT (Lookup table) mesh plots showed that TCHQ at 1 to 10 μg/mL dose-dependently reduced biofilm formation on the bottoms of polystyrene plates (Fig. 1E). Confocal microscopy showed that TCHQ dose-dependently inhibited biofilm formation, whereas dense biofilms were observed for untreated controls (Fig. 2A). Biofilm reduction was further quantified by COMSTAT analysis, which showed TCHQ at 5 and 10 μg/mL dramatically reduced average biofilm thickness, substratum coverage, and biofilm formation roughness (Fig. 2B). Specifically, mean thickness and substratum coverage were reduced by TCHQ at 5 μg/mL by more than 95% versus the untreated control, while HQ at 10 μg/mL had no effect.

### TCHQ inhibited C. albicans hyphal development.

**FIG 3:**
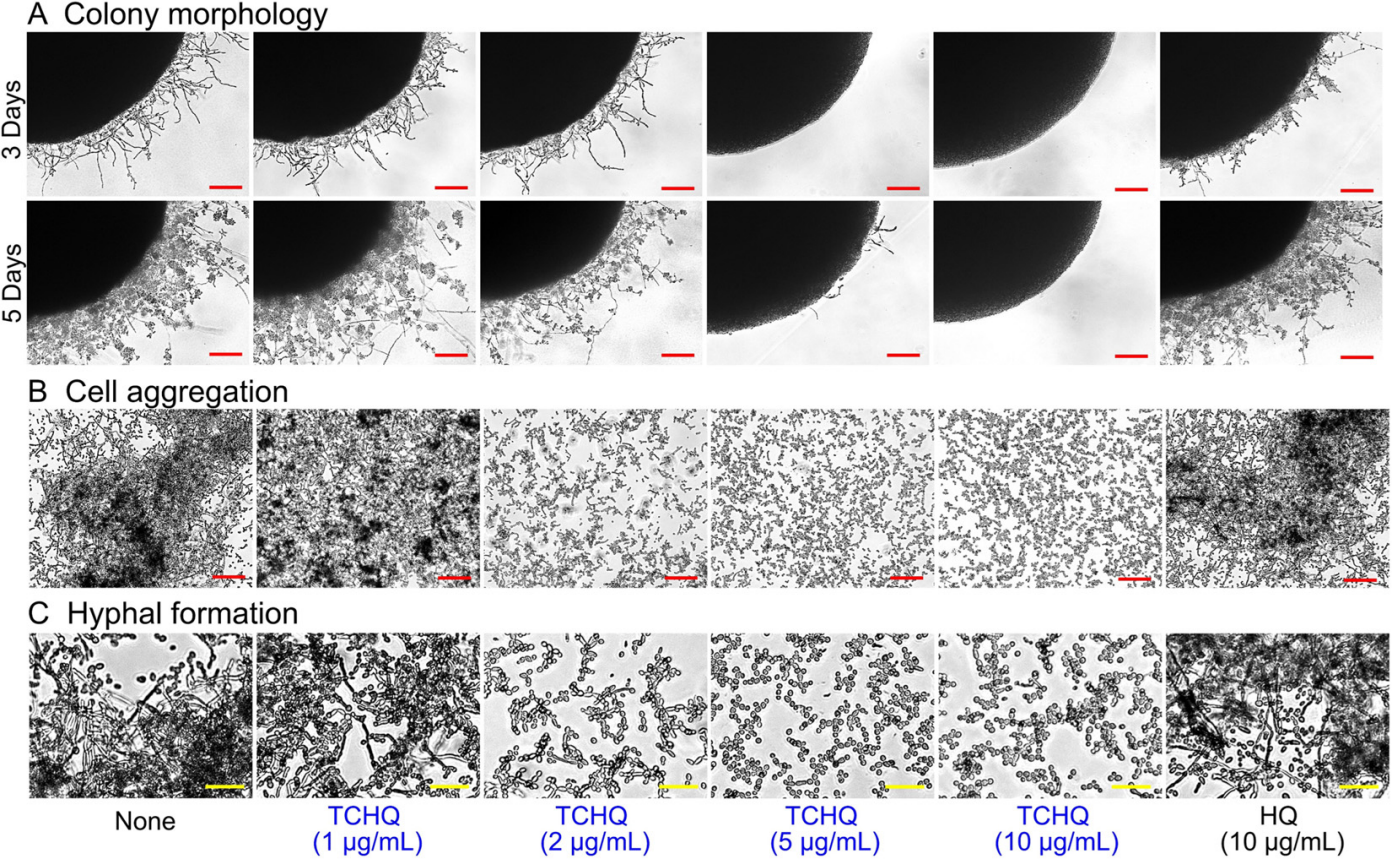
Effects of TCHQ on C. albicans morphogenesis. (A) C. albicans DAY185 morphology after cultivation on PDA solid medium for 5 days. Inhibition of cell aggregation (B) and hyphal formation (C) in PDB liquid medium. The red and yellow scale bars represent 100 and 30 μm, respectively. None, nontreated control.

**FIG 4:**
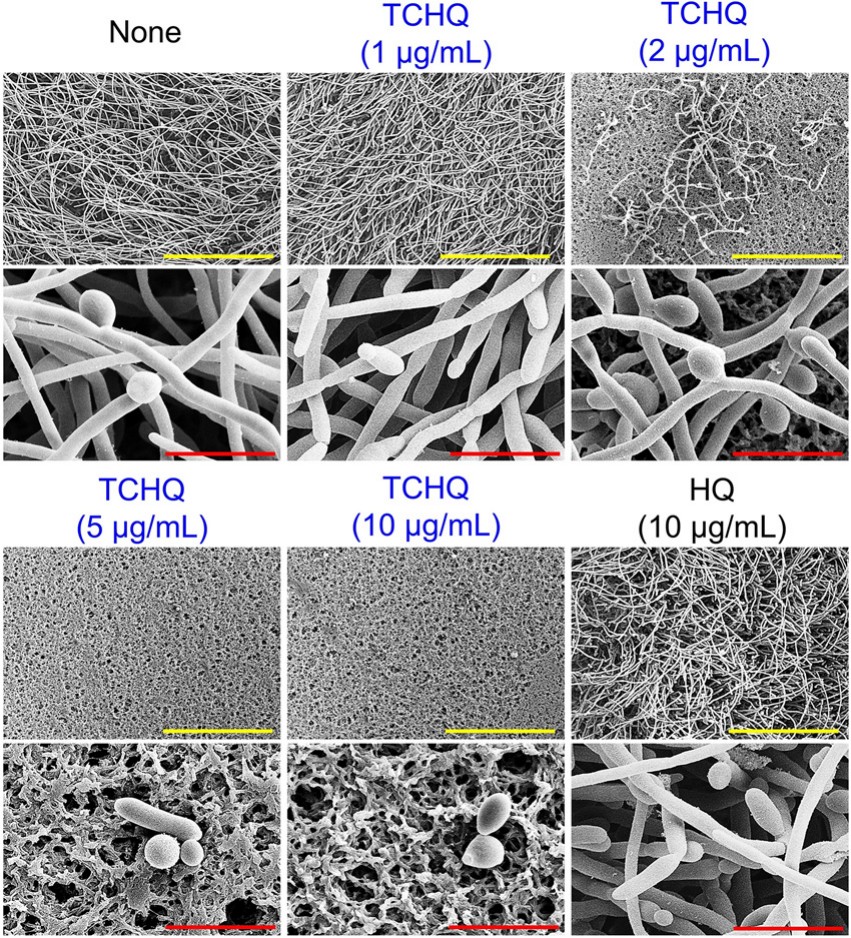
Inhibitions of hyphal growth in C. albicans biofilms by TCHQ were visualized by SEM. The yellow and red scale bars represent 100 and 10 μm, respectively. None, nontreated control.

Hyphal formation and cell aggregation are prerequisites for C. albicans biofilm formation, and thus, we performed hyphal protrusion assays on solid agar and examined cell aggregation and yeast-hyphal transition in liquid medium. In untreated controls, progressive hyphal protrusion was observed around C. albicans colonies from 3 days after plating, whereas TCHQ at 5 and 10 μg/mL abolished hyphal protrusion for 5 days (Fig. 3A). In liquid potato dextrose broth (PDB) medium, C. albicans in the untreated control produced large cell aggregates entangled by hyphae after 24 h, whereas TCHQ at 1 to 10 μg/mL inhibited filamentous growth and cell aggregation (Fig. 3B). Additionally, in cultures observed at higher magnification, untreated controls showed massive hyphal growth with few pseudohyphae and yeast cells after 24 h, but TCHQ at 2 to 10 μg/mL completely prevented hyphal growth and showed only yeast cells (Fig. 3C). SEM analysis (Fig. 4) showed that nontreated controls consisted of mixtures of hyphae and few yeast cells and that TCHQ at 5 or 10 μg/mL reduced adhesion of C. albicans and hyphal lengths and increased the proportion of yeast cells on nylon membranes. These microscopic observations revealed that TCHQ at subinhibitory concentrations inhibited yeast to hyphal transition, adhesion, and aggregation of cells and thus, inhibited biofilm formation.

### Gene expression changes in C. albicans after TCHQ treatment.

**FIG 5:**
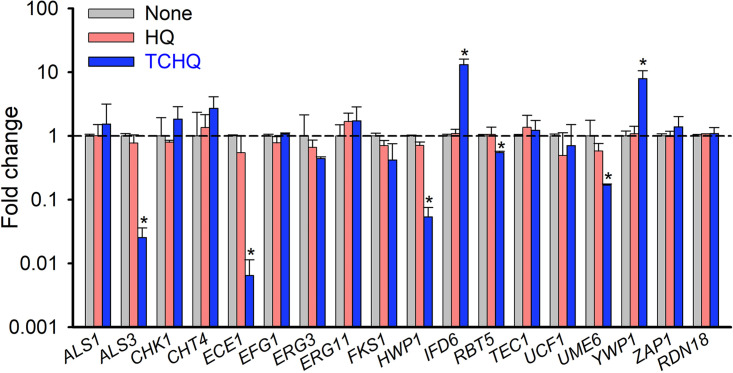
Relative transcriptional profiles of biofilm- and hyphae-related genes. C. albicans DAY185 strain was incubated in PDB medium with or without TCHQ or HQ at 5 μg/mL for 6 h, and fold changes were assessed by qRT-PCR. *RDN18* was used as the housekeeping gene. *, *P* < 0.05 versus nontreated controls.

qRT-PCR was used to study the expressions of 17 biofilm- and hyphae-related genes in C. albicans after treating C. albicans with TCHQ or HQ at 5 μg/mL for 6 h. TCHQ markedly downregulated the expressions of five hyphae-specific genes: *viz.* agglutinin-like protein *ALS3* (39-fold), hyphae-specific protein *ECE1* (155-fold), hyphal cell wall protein *HWP1* (19-fold), hyphae-associated cell wall protein *RBT5* (2-fold), and filament-specific regulator *UME6* (6-fold), and upregulated the expressions of two biofilm-related genes, alcohol dehydrogenase *IFD6* (13-fold) and yeast-form wall protein *YWP1* (8-fold), compared with untreated controls. HQ at 5 μg/mL had no effect (Fig. 5). These qRT-PCR results showed TCHQ significantly affected the expressions of some hyphae- and biofilm-related genes, supporting the inhibition of biofilm formation and hyphae development.

### TCHQ prevented C. albicans adhesion to porcine skin.

**FIG 6:**
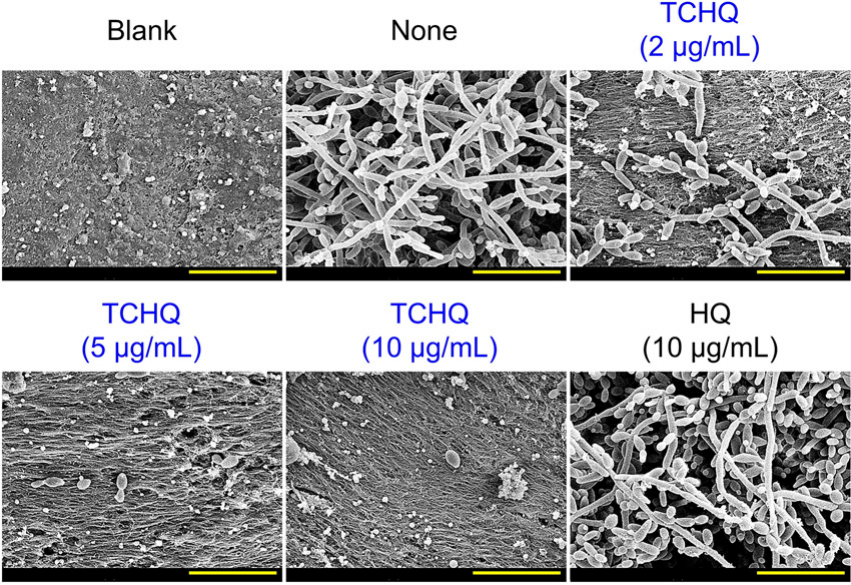
Effects of TCHQ on biofilm development by C. albicans on porcine skin. The effect of TCHQ on C. albicans attachment to porcine skin was assessed by SEM after culture for 24 h. Yellow scale bars indicate 20 μm.

Since C. albicans inhabits animal skins, we used SEM to study the ability of TCHQ to inhibit C. albicans adhesion to porcine skin. Untreated C. albicans DAY185 controls formed dense biofilms comprised of hyphal cells and few yeast cells on skin samples, whereas TCHQ dose-dependently inhibited hyphal and biofilm development (Fig. 6). For example, TCHQ at 5 μg/mL (10% of its MIC) completely prevented C. albicans attachment.

### Toxicity of HQs in a nematode model and a plant germination model.

**FIG 7:**
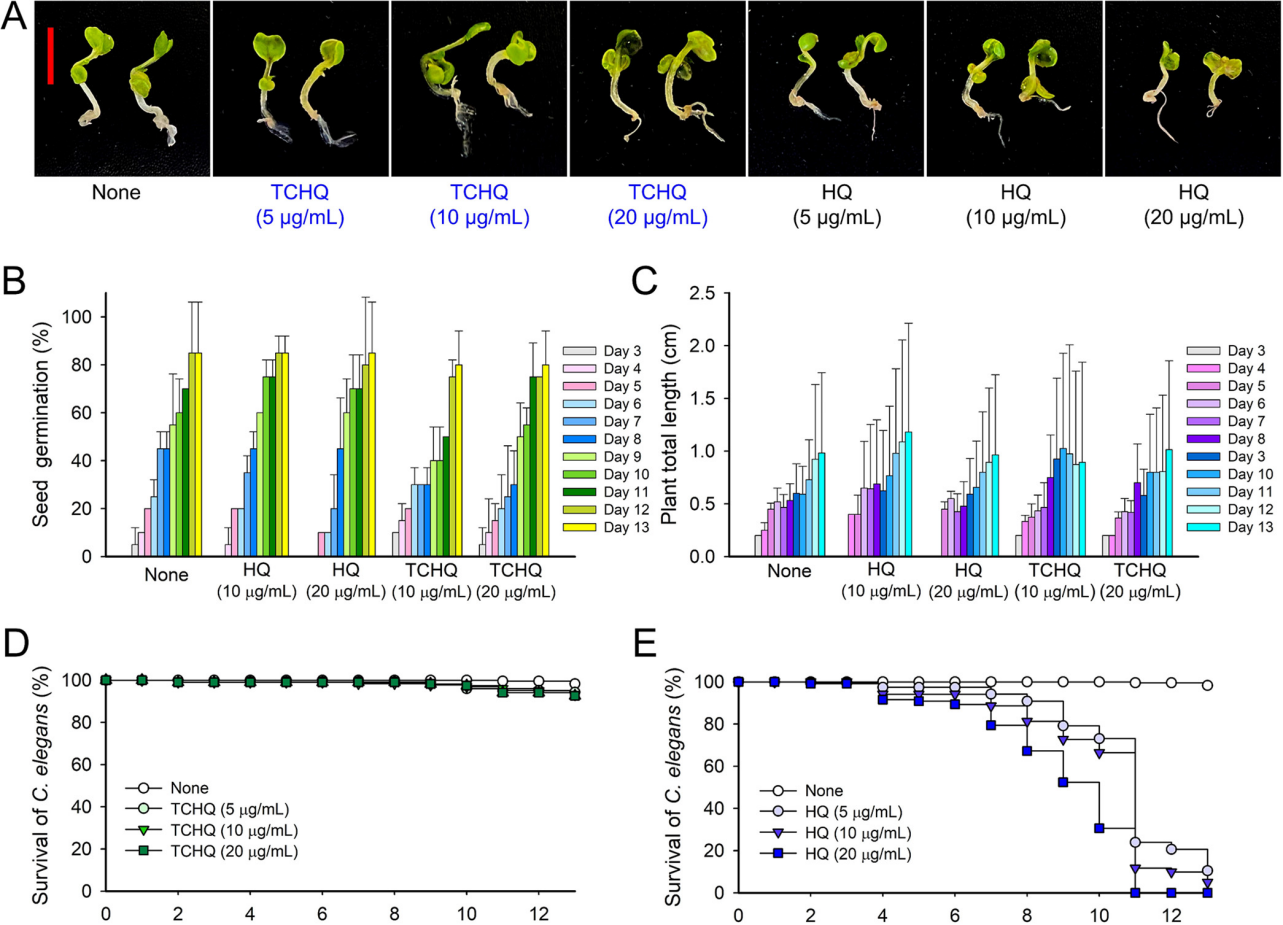
Toxicities of HQ and TCHQ in plant germination and nematode models. (A) *B. rapa* seed germination was performed using Murashige and Skoog agar medium supplemented with or without HQ or TCHQ (50 μg/mL) at 25°C. Seed germination rate (B) and plant total length (C) were analyzed for 13 days. (D and E) C. elegans survival was assessed in the presence and absence of HQ or TCHQ for 13 days.

Since the safety of HQ has been questioned (10), toxicity assessments of TCHQ and HQ were performed using a seed germination system and a C. elegans model. The seed germination rate was not affected by either TCHQ or HQ at tested concentrations (5, 10, and 20 μg/mL) (Fig. 7B), and plant heights were not much different in TCHQ and HQ for 13 days (Fig. 7A–C). The effects of TCHQ and HQ were also investigated on the Caenorhabditis elegans survival. We found that TCHQ was less toxic to C. elegans than HQ (Fig. 6D and E). For example, most nematodes survived after treatment with TCHQ at 20 μg/mL for 13 days, whereas the majority died after treatment with HQ at 5 to 20 μg/mL for the same amount of days. These results indicate that TCHQ in its active antibiofilm range (5 to 20 μg/mL) was not toxic to B. rapa growth and nematode C. elegans. Additional toxicity assay was performed with all 18 HQs at higher concentrations (20, 50, 100, and 200 μg/mL) and found that most HQs at high concentrations significantly decreased the survival of the nematode (Fig. S2). Particularly, 2,3-dimethylhydroquinone, 2,6-dimethylhydroquinone, 2-methoxyhydroquinone, and trimethylhydroquinone at 100 μg/mL were toxic to the nematode.

### ADME profiling of HQ and TCHQ.

Absorption, distribution, metabolism, and excretion (ADME) profiles of HQ and TCHQ were also evaluated. Neither HQ nor TCHQ violated Lipinski’s rule of five. Both had acceptable skin and brain barrier permeabilities and human intestinal adsorptions, did not exhibit acute fish toxicity, and were noncarcinogenic to mice. Full ADME profiles are presented in Table S1.

## DISCUSSION

**FIG 8 fig8:**
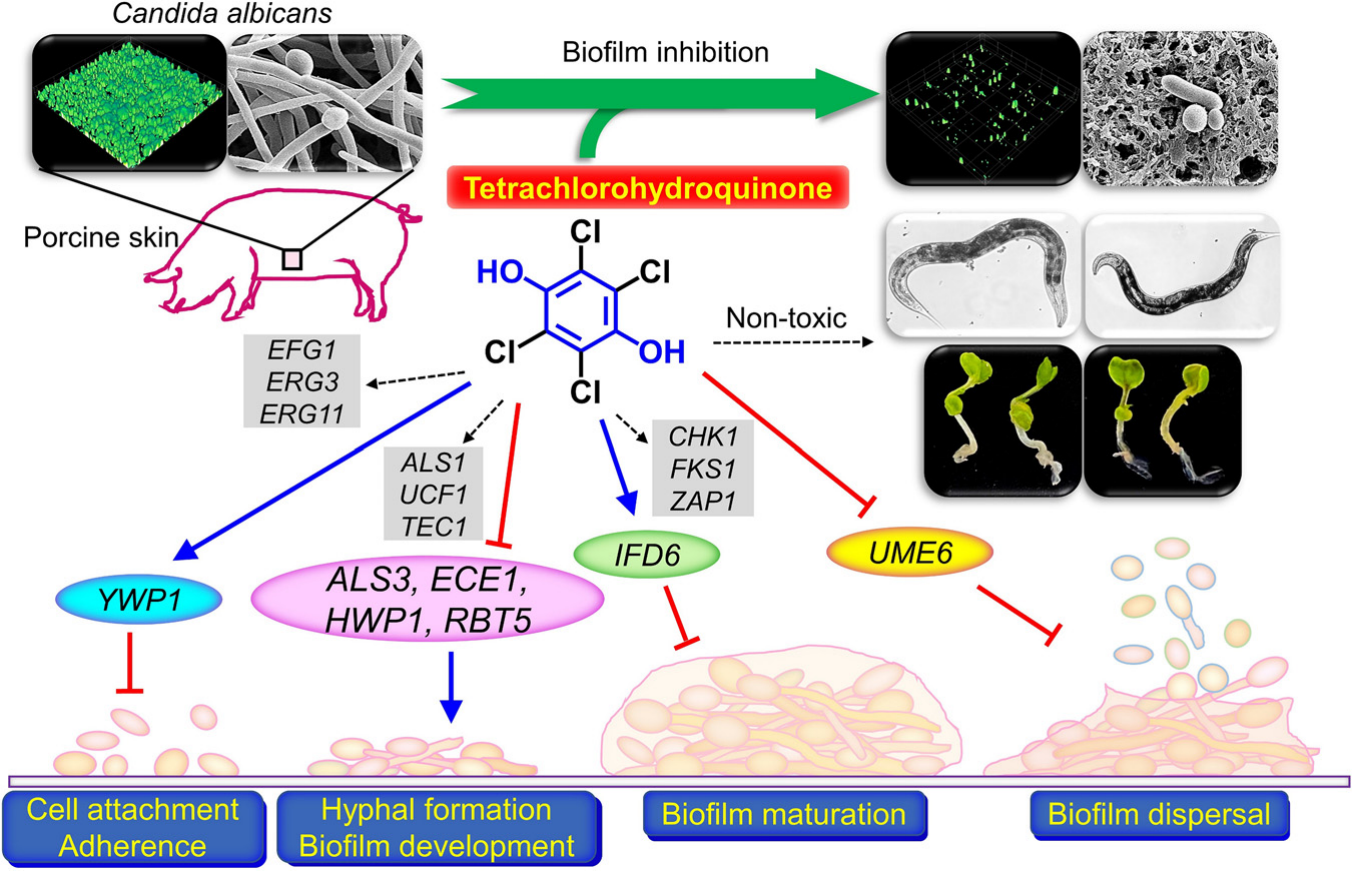
Diagram of the putative mechanism of HQs in C. albicans. → indicates upregulation of gene expression or positively affecting a phenotype, ⊦ indicates downregulation of gene expression or negatively affecting a phenotype, and black dotted lines indicate no change/no effect.

We report the biofilm-inhibitory abilities of a series of HQs against a drug-resistant C. albicans strain. In particular, the most active hydroquinone TCHQ at subinhibitory concentrations (2 to 10 μg/mL) significantly inhibited biofilm formation, cell aggregation, and hyphal development. Notably, TCHQ also significantly affected the gene expressions of seven biofilm-related and hyphae-specific genes and exhibited little toxicity in C. elegans and *B. rapa* seed germination[Fig fig8].

Hydroquinone (HQ) and its derivatives have been reported to exhibit antimicrobial activities against drug-resistant Staphylococcus aureus and Pseudomonas aeruginosa (11–13). However, the mechanisms responsible have not been determined, and little is known of their antibiofilm activities. Previously, it was reported that several anthraquinones inhibit biofilm and hyphal formation by C. albicans and exhibit only mild animal toxicity (9). Also, chloramine T trihydrate with a benzene ring showed antifungal and antibiofilm activities against *Candida* spp. (14), and 2-bromo-2-chloro-2-(4-chlorophenylsulfonyl)-1-phenylethanon, which contains two benzene rings, exhibited antifungal activity against *Candida* spp. (15). Various halogen derivatives of biologically active compounds have been reported to enhance drug activities (16, 17). In this study, HQ and tetrafluorohydroquinone showed no or little effect on C. albicans, whereas dibromohydroquinone and TCHQ exhibited significant antifungal and antibiofilm activities (Table 1). Although speculative, the addition of electron-withdrawing halogens, such as two bromine atoms in 2,5-dibromohydroquinone and four Cl-atoms in TCHQ, may increase electrophilicity and increase antifungal and antibiofilm activities. Notably, we found TCHQ was less toxic than HQ in a nematode model and a plant model (Fig. 7). Notably, TCHQ at 5 μg/mL, markedly inhibited *Candida* biofilm and hyphal development by 90%, and this concentration was 10-fold lower than its MIC for planktonic cells (Fig. 2 and 3). Unlike antifungal agents that inhibit cell growth, TCHQ at low concentrations (2 to 10 μg/mL) suppressed biofilm and hyphal formation development and only showed antifungal activity at concentrations > 50 μg/mL, which indicates TCHQ may be less prone to the generation of drug resistance. HQ is a well-known antihyperpigmentation agent with a history of more 40 years, but the toxicity of HQs has been of concern, as HQ at high concentrations induced a process of carcinogenesis (10). Also, TCHQ showed its toxicity against mice with 370 mg/kg of oral LD_50_ (10) and trout liver cells with 15.85 μM (3.7 μg/mL) of MTT_50_ (18). While this study provides preliminary toxicity data with plant and nematode models (Fig. 7 and Fig. S2), more rigorous toxicological study would be further verified with animal models. Also, anthraquinones (9) and HQs in this study showed strong antibiofilm activity and mild cytotoxicity; their applications are limited by poor water solubilities. To address this limitation, nanoparticle-based drug carriers conjugated with 1,2-dihydroxyanthraquinone (alizarin) have been used as C. albicans antibiofilm agents (19) since the nanodrug loading system can slow the release of drugs, reduce cell biological toxicity, and increase drug efficacy.

Our qRT-PCR studies showed that TCHQ at 5 μg/mL significantly repressed the gene expression of *ALS3*, *ECE1*, *HWP1*, *RBT1*, and *UME6* but induced those of *IFD6* and *YWP1* in C. albicans cells (Fig. 5). *ALS3* is essential for hyphal development (20), *ECE1* encodes a protein involved in hyphal cell elongation and biofilm formation (21), *HWP1* (hyphal cell wall protein, also known as *ECE2*) is essential for hyphal development and biofilm formation (22), *RBT5* (fungal-type cell wall protein) expression is induced during hyphal growth (23), and *UME6* is a filament-specific regulator of hyphal extension and maintenance (24). On the other hand, *IFD6* is an inhibitor of biofilm matrix production (25) and *YWP1* encodes yeast form wall protein 1, and thus, is an indicator of hyphal inhibition (26, 27). Experimentally, TCHQ inhibited hyphal protrusion on solid agar, *Candida* cell aggregation, and hyphal development in liquid media (Fig. 3), which is in line with its observed antibiofilm activity. Taken together, our results suggest that TCHQ inhibits cell adhesion, biofilm formation, and hyphal development by downregulating hyphae-specific genes and upregulating inhibitors of hyphal growth (Fig. 5).

Furthermore, our qRT-PCR results support those of several previous studies on the effects of antibiofilm agents on C. albicans. For example, two anthraquinones (alizarin and chrysazin) (9) and 7-benzyloxyindole (28) inhibited biofilm and hyphal formation by downregulating the gene expressions of *ALS3*, *ECE1*, *HWP1*, and *RBT1*, linoleic acid repressed the gene expressions of *ECE1*, *HWP1*, *RBT1* and *UME6* (29), heptanoic and nonanoic acid repressed the expressions of *ALS3*, *ECE1*, *HWP1*, and *UME6* (30), nepodin or propolin D repressed those of *ECE1*, *HGT10*, *HWP1*, and *UME6* (31, 32), and saw palmetto oil repressed the gene expressions of *HWP1* and *UME6* (33). However, although the transcriptional changes induced by these antibiofilm compounds are similar, the exact genes or proteins targeted by these compounds have yet to be determined.

The emergence of multidrug-resistant *Candida* strains has promoted the development of antibiofilm and antivirulence agents. The present study shows TCHQ inhibits C. albicans biofilm formation by repressing hyphal development, and that TCHQ exhibits minimal chemical toxicity against a C. elegans and the seeds of *B. rapa* (Fig. 8). Further *in vivo* experiments using a mouse model are required to confirm the efficacy of TCHQ against drug-resistant C. albicans. Moreover, other HQ derivatives could offer a basis for the design of potent anti-*Candida* agents.

## MATERIALS AND METHODS

### Strains and reagents.

C. albicans strains DAY185 (fluconazole resistant MIC > 1,024 μg/mL) and ATCC 10231 (fluconazole sensitive) were obtained from the Korean Culture Centre for Microorganisms (KCCM) and the American Type Culture Collection (ATCC), respectively. C. albicans strains were subcultured in potato dextrose agar (PDA) or potato dextrose broth (PDB). PDA plates were placed at 37°C for 48 h, and a single fresh colony was used to inoculate into 25 mL of PDB in 250-mL flat-bottomed flasks and cultivated overnight at 37°C. The 18 compounds tested were as follows: 2,5-bis(1,1,3,3-tetramethylbutyl) hydroquinone, chlorohydroquinone, 2,5-dibromohydroquinone, 2,3-dicyanohydroquinone, 2,3-dimethylhydroquinone, 2,6-dimethylhydroquinone, 2,5-di-tert-butylhydroquinone, hydroquinone (HQ), hydroquinone monobenzyl ether, hydroquinone-o,ò-diacetic acid, methylhydroquinone, 2-methoxyhydroquinone, 4-methoxyphenol, tert-butylhydroquinone, tetrachlorohydroquinone (TCHQ), tetrafluorohydroquinone, 4-((tetrahydro-2H-pyran-2-yl)oxy)phenol, and trimethylhydroquinone (Table 1). All were purchased from Sigma-Aldrich (St. Louis, MO, USA) or Combi-blocks (San Diego, CA, USA), and dissolved in dimethyl sulfoxide (DMSO), the concentration of which did not exceed 0.1% (vol/vol) in any experiment. To assess cell growths, culture turbidities were determined at 620 nm using a spectrophotometer (Multiskan EX microplate reader; Thermo Fisher Scientific, Waltham, MA, USA) after cultivation for 24 h at 37°C. MICs were defined based on the Clinical and Laboratory Standards Institute (CLSI) broth dilution method (34), using 96-well polystyrene plates (SPL Life Sciences, Pocheon, Republic of Korea). Briefly, C. albicans cells were cultured overnight in PDB, diluted to ~10^5^ cells/mL, added to the wells of a 96-well plate containing different concentrations (wt/vol) of the tested HQ derivatives, and incubated for 24 h at 37°C. MIC was decided as the lowest concentration inhibiting microbial growth by 80% as evaluated by spectrophotometry (620 nm) and colony counting.

### Assays of biofilm inhibition.

C. albicans biofilms were prepared on 96-well polystyrene plates, as previously reported (33). Briefly, overnight cultures of C. albicans cells were inoculated into fresh PDB at a beginning turbidity of 0.1 at 600 nm and cultivated with or without test HQ derivatives at various concentrations for 24 h without agitation at 37°C. Biofilm formation was quantified after washing plates three times with distilled H_2_O to remove free-floating cells. Biofilms were incubated with 0.1% crystal violet for 20 min, cleaned three times with distilled H_2_O, and then the crystal violet in biofilm cells was extracted using 95% ethanol. Absorbances were measured using the Multiskan EX microplate reader at 570 nm, and results are expressed as the averages of at least six replicates.

### Assays of C. albicans hyphal development.

To investigate the colony morphology of C. albicans on solid PDA medium, a glycerol stock was used to streak cells on PDA plates containing HQs and incubated for 5 days at 37°C. Colony morphologies of live cells were observed under an optical microscope (iRiS Digital Cell Imaging System, Anyang, Republic of Korea).

Cell aggregation was analyzed as previously described (35). Briefly, C. albicans cells were inoculated into 3 mL of PDB at a density of ~10^5^ CFU/mL in 14-mL test tubes with or without HQs and incubated under dark for 24 h at 37°C without shaking. Well-mixed cell cultures (0.1 mL) were then transferred to glass-bottomed dishes and observed. Hyphal formation and cell aggregation were observed in bright field using the iRiS Digital Cell Imaging System at magnifications of 4× and 10×. At least, four independent experiments were conducted.

### Microscopic observations of C. albicans biofilm formation.

To observe biofilm formation, biofilms were produced, as mentioned above, over 24 h at 37°C. Free-floating cells were then removed by gentle washing with distilled H_2_O three times and biofilms were visualized by live imaging microscopy using the iRiS Digital Cell Imaging System (Logos BioSystems). Biofilm images were generated as color-coded 2D and 3D pictures using ImageJ.

Also, C. albicans cells were cultivated in 96-well polystyrene plates (SPL Life Sciences) without shaking in the absence or presence of HQs. Free-floating cells were then removed by washing with sterile phosphate-buffered saline (PBS) three times. C. albicans biofilm cells were incubated with carboxyfluorescein diacetate succinimidyl ester (a cell-permeable dye; Thermo Fisher Scientific, Waltham, MA, USA) (36), which becomes highly fluorescent when it loses its acetyl groups due to intracellular esterase activity. Biofilms on plate bottoms were visualized using a 488-nm argon laser (emission wavelength 500 to 550 nm) of a confocal laser microscope (Nikon Eclipse Ti, Tokyo, Japan) equipped with a 20× objective (37). Color confocal images were constructed using NIS-Elements C version 3.2 (Nikon Eclipse). At least 12 random positions in two independent cultures were analyzed per experiment.

### Examination of hyphae by scanning electron microscopy (SEM).

SEM was used to investigate hyphal formation, as previously described (38). Briefly, a sterile nylon membrane (Whatman, Maidstone, UK) was cut into 0.4 × 0.4 cm pieces and single pieces were set in the wells of 96-well plates having 300 μL of cell suspension of turbidity 0.1 at 600 nm. Cells were incubated in the presence or absence (untreated controls) of HQs for 24 h at 37°C without shaking. Biofilm cells were then fixed with a glutaraldehyde (2.5%) and formaldehyde (2%) mixture for 24 h at 4°C, postfixed in osmium tetroxide (1% OsO_4_), and dehydrated using a series of ethanol (50, 70, 80, 90, 95, and 100%) followed by isoamyl acetate. After critical-point drying, cells on nylon membranes were examined under an S-4800 field emission scanning electron microscope (FE-SEM, Hitachi, Japan) at a voltage of 10 kV and magnifications ranging from 500× to 5,000×.

### Quantitative real-time PCR (qRT-PCR).

For transcriptional analysis of C. albicans genes, C. albicans was inoculated into 20 mL of fresh sterile PDB in 250-mL flat-bottomed flasks at a beginning optical density at 600 nm (OD_600_) of ~0.1, and then cultivated for 6 h at 37°C without shaking in the presence or absence of HQ or TCHQ (5 μg/mL). RNase inhibitor (RNAlater, Ambion, TX, USA) was added and mixed gently to prevent RNA degradation. Total RNA was isolated and purified using a hot acidic phenol method, as previously described (39), and purified using an RNeasy minikit (Qiagen, Hilden, Germany). qRT-PCR was used to investigate the transcription levels of hyphae- and biofilm-related genes (*ALS1*, *ALS3*, *CHK1*, *CHT4*, *ECE1*, *EFG1*, *ERG3*, *ERG11*, *FKS1*, *HWP1 [also called ECE2]*, *IFD6*, *RBT5*, *TEC1*, *UCF1*, *UME6*, *YWP1*, and *ZAP1*). Gene-specific primers were used, and *RDN18* was used as a housekeeping control (Table S2). The qRT-PCR method used has been previously described (30). qRT-PCR was carried out using a SYBR green qPCR master mix (Applied Biosystems, Foster City, CA, USA) and a StepOne real-time PCR system (Applied Biosystems) on two independent cultures with four reactions.

### Biofilm inhibition analysis on porcine skin.

The assay used was a modification of a method devised by Lee et al. (40, 41). Briefly, freshly frozen porcine skin (Korea Federation of Livestock Cooperatives, Seoul, Republic of Korea) was purchased and stored at −80°C until required. Skin was sterilized before use by immersing 0.6 × 0.6 cm pieces sequentially in 70% ethanol and 10% bleach solution for 30 min. Pieces were then washed three times with sterile H_2_O for 10 min. C. albicans cells (10^5^ CFU/mL) in PDB were then added to the wells of a 12-well plate containing one piece of porcine skin per well and incubated with or without HQ and TCHQ for 24 h at 37°C. SEM analysis was performed as described above. Two independent samples were analyzed.

### Seed germination assay.

To analyze the toxicity and effect on the germination and plant growth of Chinese cabbage (*B. rapa*), seeds of *B. rapa* were soaked in sterile distilled H_2_O for 16 h and rinsed with H_2_O three times. To sterilize the surface of seeds, seeds were incubated sequentially in 95% ethanol and 3% sodium hypochlorite for 15 min at room temperature (25°C) and rinsed with sterile distilled H_2_O three times. Next, 10 seeds per plate were placed on soft agar Murashige and Skoog plates constituting 0.7% agar and 0.86 g/L Murashige and Skoog (MS) and were incubated at room temperature for 13 days. Seed germination percentages and total length of plant were measured. Four independent experiments were performed for each concentration.

### Chemical toxicity assays in the nematode model.

To investigate the chemical toxicity of HQ and TCHQ, we used C. elegans strain *fer-15*(*b26*); *fem-1*(*hc17*), as previously described (30). Briefly, synchronized adult nematodes were washed twice with M9 buffer (3 g/L KH_2_PO_4_, 6 g/L Na_2_HPO_4_, 5 g/L NaCl, 1 mM MgSO_4_) before starting experiments. About 40 worms were placed into each well of 96-well plates containing M9 buffer (200 μL) with HQ and TCHQ (5, 10 and 20 μg/mL). Plates were then incubated for 13 days at 25°C without agitation. Four independent experiments were performed in triplicate. Results are expressed as percentages of live worms, as determined by responses to LED lights for 20 to 30s using an iRiS Digital Cell Imaging System (Logos Bio Systems) after incubation.

### Predictions of absorption, distribution, metabolic, and excretion properties (ADME).

The drug-like properties of HQ and TCHQ were evaluated using ADME software (42). Online webservers, *viz.* PreADMET (https://preadmet.qsarhub.com/), Molinspiration (https://www.molinspiration.com/), and GUSAR (http://www.way2drug.com/gusar/) were accessed on 3 June 2022. According to Lipinski’s rule of five, an orally active drug should have a molecular weight of ≤500 g/mol, a Log P of ≤5, ≤5 hydrogen bond-donating atoms, ≤10 hydrogen-bond accepting atoms, and an octanol-water partition coefficient of ≤140 Å2 (43).

### Statistical analysis.

The analysis was conducted using one-way ANOVA subsequently followed by Dunnett’s test in SPSS version 23 (SPSS Inc., Chicago, IL, USA). *P* values of <0.05 were treated significant. Asterisks are employed to denote significant differences between untreated and treated samples, and results are presented as means ± standard deviations. Sample replication numbers are provided above.

## References

[1] Ramage G, Saville SP, Thomas DP, Lopez-Ribot JL. 2005. *Candida* biofilms: an update. Eukaryot Cell 4:633–638. doi:10.1128/EC.4.4.633-638.2005. 15821123 PMC1087806

[2] Sudbery PE. 2011. Growth of *Candida albicans* hyphae. Nat Rev Microbiol 9:737–748. doi:10.1038/nrmicro2636. 21844880

[3] Cavalheiro M, Teixeira MC. 2018. *Candida* biofilms: threats, challenges, and promising strategies. Front Med (Lausanne) 5:28. doi:10.3389/fmed.2018.00028. 29487851 PMC5816785

[4] Mah TF, O'Toole GA. 2001. Mechanisms of biofilm resistance to antimicrobial agents. Trends Microbiol 9:34–39. doi:10.1016/S0966-842X(00)01913-2. 11166241

[5] Staniszewska M. 2020. Virulence factors in *Candida* species. Curr Protein Pept Sci 21:313–323. doi:10.2174/1389203720666190722152415. 31544690

[6] Clatworthy AE, Pierson E, Hung DT. 2007. Targeting virulence: a new paradigm for antimicrobial therapy. Nat Chem Biol 3:541–548. doi:10.1038/nchembio.2007.24. 17710100

[7] Rasko DA, Sperandio V. 2010. Anti-virulence strategies to combat bacteria-mediated disease. Nat Rev Drug Discov 9:117–128. doi:10.1038/nrd3013. 20081869

[8] Dickey SW, Cheung GYC, Otto M. 2017. Different drugs for bad bugs: antivirulence strategies in the age of antibiotic resistance. Nat Rev Drug Discov 16:457–471. doi:10.1038/nrd.2017.23. 28337021 PMC11849574

[9] Manoharan RK, Lee J-H, Kim Y-G, Lee J. 2017. Alizarin and chrysazin inhibit biofilm and hyphal formation by *Candida albicans*. Front Cell Infect Microbiol 7:447. doi:10.3389/fcimb.2017.00447. 29085811 PMC5650607

[10] Nordlund JJ, Grimes PE, Ortonne JP. 2006. The safety of hydroquinone. J Eur Acad Dermatol Venereol 20:781–787. doi:10.1111/j.1468-3083.2006.01670.x. 16898897

[11] Ma C, He N, Zhao Y, Xia D, Wei J, Kang W. 2019. Antimicrobial mechanism of hydroquinone. Appl Biochem Biotechnol 189:1291–1303. doi:10.1007/s12010-019-03067-1. 31254228

[12] Hammoud Mahdi D, Hubert J, Renault JH, Martinez A, Schubert A, Engel KM, Koudogbo B, Vissiennon Z, Ahyi V, Nieber K, Vissiennon C. 2020. Chemical profile and antimicrobial activity of the fungus-growing termite strain *Macrotermes Bellicosus* used in traditional medicine in the republic of benin. Molecules 25:5015. doi:10.3390/molecules25215015. 33138110 PMC7662623

[13] Jeyanthi V, Velusamy P, Kumar GV, Kiruba K, Aeruginosa P. 2021. Effect of naturally isolated hydroquinone in disturbing the cell membrane integrity of *Pseudomonas aeruginosa* MTCC 741 and *Staphylococcus aureus* MTCC 740. Heliyon 7:e07021. doi:10.1016/j.heliyon.2021.e07021. 34036196 PMC8134992

[14] Ferreira GLS, Rosalen PL, Peixoto LR, Perez A, Carlo FGC, Castellano LRC, Lima JM, Freires IA, Lima EO, Castro RD. 2017. Antibiofilm activity and mechanism of action of the disinfectant chloramine T on *Candida* spp., and its toxicity against human cells. Molecules 22:1527. doi:10.3390/molecules22091527. 28926981 PMC6151619

[15] Staniszewska M, Bondaryk M, Wieczorek M, Estrada-Mata E, Mora-Montes HM, Ochal Z. 2016. Antifungal effect of novel 2-bromo-2-chloro-2–(4-chlorophenylsulfonyl)-1-phenylethanone against *Candida* strains. Front Microbiol 7:1309. doi:10.3389/fmicb.2016.01309. 27610100 PMC4996825

[16] Wilcken R, Liu XR, Zimmermann MO, Rutherford TJ, Fersht AR, Joerger AC, Boeckler FM. 2012. Halogen-enriched fragment libraries as leads for drug rescue of mutant p53. J Am Chem Soc 134:6810–6818. doi:10.1021/ja301056a. 22439615 PMC3789257

[17] Hernandes MZ, Cavalcanti SM, Moreira DR, de Azevedo Junior WF, Leite AC. 2010. Halogen atoms in the modern medicinal chemistry: hints for the drug design. Curr Drug Targets 11:303–314. doi:10.2174/138945010790711996. 20210755

[18] Pietsch C, Hollender J, Dorusch F, Burkhardt-Holm P. 2014. Cytotoxic effects of pentachlorophenol (PCP) and its metabolite tetrachlorohydroquinone (TCHQ) on liver cells are modulated by antioxidants. Cell Biol Toxicol 30:233–252. doi:10.1007/s10565-014-9283-4. 24996998

[19] Ramasamy M, Nanda SS, Lee J-H, Lee J. 2020. Construction of alizarin conjugated graphene oxide composites for inhibition of *Candida albicans* biofilms. Biomolecules 10:565. doi:10.3390/biom10040565. 32272698 PMC7226399

[20] Argimon S, Wishart JA, Leng R, Macaskill S, Mavor A, Alexandris T, Nicholls S, Knight AW, Enjalbert B, Walmsley R, Odds FC, Gow NA, Brown AJ. 2007. Developmental regulation of an adhesin gene during cellular morphogenesis in the fungal pathogen *Candida albicans*. Eukaryot Cell 6:682–692. doi:10.1128/EC.00340-06. 17277173 PMC1865654

[21] Nobile CJ, Andes DR, Nett JE, Smith FJ, Yue F, Phan QT, Edwards JE, Filler SG, Mitchell AP. 2006. Critical role of Bcr1-dependent adhesins in *C. albicans* biofilm formation *in vitro* and *in vivo*. PLoS Pathog 2:e63. doi:10.1371/journal.ppat.0020063. 16839200 PMC1487173

[22] Nobile CJ, Nett JE, Andes DR, Mitchell AP. 2006. Function of *Candida albicans* adhesin Hwp1 in biofilm formation. Eukaryot Cell 5:1604–1610. doi:10.1128/EC.00194-06. 17030992 PMC1595337

[23] Braun BR, Head WS, Wang MX, Johnson AD. 2000. Identification and characterization of TUP1-regulated genes in *Candida albicans*. Genetics 156:31–44. doi:10.1093/genetics/156.1.31. 10978273 PMC1461230

[24] Banerjee M, Uppuluri P, Zhao XR, Carlisle PL, Vipulanandan G, Villar CC, Lopez-Ribot JL, Kadosh D. 2013. Expression of *UME6*, a key regulator of *Candida albicans* hyphal development, enhances biofilm formation via Hgc1-and Sun41-dependent mechanisms. Eukaryot Cell 12:224–232. doi:10.1128/EC.00163-12. 23223035 PMC3571304

[25] Nobile CJ, Nett JE, Hernday AD, Homann OR, Deneault JS, Nantel A, Andes DR, Johnson AD, Mitchell AP. 2009. Biofilm matrix regulation by *Candida albicans* Zap1. PLoS Biol 7:e1000133. doi:10.1371/journal.pbio.1000133. 19529758 PMC2688839

[26] Granger BL, Flenniken ML, Davis DA, Mitchell AP, Cutler JE. 2005. Yeast wall protein 1 of *Candida albicans*. Microbiology (Reading) 151:1631–1644. doi:10.1099/mic.0.27663-0. 15870471

[27] Finkel JS, Mitchell AP. 2011. Genetic control of *Candida albicans* biofilm development. Nat Rev Microbiol 9:109–118. doi:10.1038/nrmicro2475. 21189476 PMC3891587

[28] Manoharan RK, Lee J-H, Lee J. 2018. Efficacy of 7-benzyloxyindole and other halogenated indoles to inhibit *Candida albicans* biofilm and hyphal formation. Microb Biotechnol 11:1060–1069. doi:10.1111/1751-7915.13268. 29656577 PMC6196399

[29] Kim Y-G, Lee J-H, Park JG, Lee J. 2020. Inhibition of *Candida albicans* and *Staphylococcus aureus* biofilms by centipede oil and linoleic acid. Biofouling 36:126–137. doi:10.1080/08927014.2020.1730333. 32093497

[30] Lee J-H, Kim Y-G, Khadke SK, Lee J. 2021. Antibiofilm and antifungal activities of medium-chain fatty acids against *Candida albicans* via mimicking of the quorum-sensing molecule farnesol. Microb Biotechnol 14:1353–1366. doi:10.1111/1751-7915.13710. 33252828 PMC8313291

[31] Lee J-H, Kim Y-G, Khadke SK, Yamano A, Watanabe A, Lee J. 2019. Inhibition of biofilm formation by *Candida albicans* and polymicrobial microorganisms by nepodin via hyphal-growth suppression. ACS Infect Dis 5:1177–1187. doi:10.1021/acsinfecdis.9b00033. 31055910

[32] Lee J-H, Kim Y-G, Khadke SK, Yamano A, Woo J-T, Lee J. 2019. Antimicrobial and antibiofilm activities of prenylated flavanones from *Macaranga tanarius*. Phytomedicine 63:153033. doi:10.1016/j.phymed.2019.153033. 31352284

[33] Kim Y-G, Lee J-H, Park S, Kim S, Lee J. 2022. Inhibition of polymicrobial biofilm formation by saw palmetto oil, lauric acid and myristic acid. Microb Biotechnol 15:590–602. doi:10.1111/1751-7915.13864. 34156757 PMC8867970

[34] Alastruey-Izquierdo A, Melhem MS, Bonfietti LX, Rodriguez-Tudela JL. 2015. Susceptibility test for fungi: clinical and laboratorial correlations in medical mycology. Rev Inst Med Trop Sao Paulo 57:57–64. doi:10.1590/S0036-46652015000700011. PMC4711191 26465371

[35] Zelante T, Iannitti RG, De Luca A, Arroyo J, Blanco N, Servillo G, Sanglard D, Reichard U, Palmer GE, Latge JP, Puccetti P, Romani L. 2012. Sensing of mammalian IL-17A regulates fungal adaptation and virulence. Nat Commun 3:683. doi:10.1038/ncomms1685. 22353714

[36] Weston SA, Parish CR. 1990. New fluorescent dyes for lymphocyte migration studies. Analysis by flow cytometry and fluorescence microscopy. J Immunol Methods 133:87–97. doi:10.1016/0022-1759(90)90322-m. 2212694

[37] Kim Y-G, Lee J-H, Kim CJ, Lee JC, Ju YJ, Cho MH, Lee J. 2012. Antibiofilm activity of *Streptomyces* sp. BFI 230 and *Kribbella* sp. BFI 1562 against *Pseudomonas aeruginosa*. Appl Microbiol Biotechnol 96:1607–1617. doi:10.1007/s00253-012-4225-7. 22722911

[38] Kim Y-G, Lee J-H, Park S, Lee J. 2022. The anticancer agent 3,3’-diindolylmethane inhibits multispecies biofilm formation by acne-causing bacteria and *Candida albicans*. Microbiol Spectr 10:e0205621. doi:10.1128/spectrum.02056-21. 35107361 PMC8809333

[39] Amin-ul Mannan M, Sharma S, Ganesan K. 2009. Total RNA isolation from recalcitrant yeast cells. Anal Biochem 389:77–79. doi:10.1016/j.ab.2009.03.014. 19302974

[40] Lee J-H, Kim Y-G, Park S, Hu L, Lee J. 2022. Phytopigment alizarin inhibits multispecies biofilm development by *Cutibacterium acnes*, *Staphylococcus aureus*, and *Candida albicans*. Pharmaceutics 14:1047. doi:10.3390/pharmaceutics14051047. 35631633 PMC9143108

[41] Lee J-H, Kim Y-G, Lee J. 2022. Inhibition of *Staphylococcus aureus* biofilm formation and virulence factor production by petroselinic acid and other unsaturated C18 fatty acids. Microbiol Spectr 10:e0133022. doi:10.1128/spectrum.01330-22. 35647620 PMC9241682

[42] Daina A, Michielin O, Zoete V. 2017. SwissADME: a free web tool to evaluate pharmacokinetics, drug-likeness and medicinal chemistry friendliness of small molecules. Sci Rep 7:42717. doi:10.1038/srep42717. 28256516 PMC5335600

[43] Lipinski CA, Lombardo F, Dominy BW, Feeney PJ. 2001. Experimental and computational approaches to estimate solubility and permeability in drug discovery and development settings. Adv Drug Deliv Rev 46:3–26. doi:10.1016/s0169-409x(00)00129-0. 11259830

