# Supplementary material for: Hydroquinones Including Tetrachlorohydroquinone Inhibit Candida albicans Biofilm Formation by Repressing Hyphae-Related Genes
Source: Microbiol Spectr. 2022 Oct 3;10(5):e02536-22. doi: 10.1128/spectrum.02536-22 (PMC9602536; doi:10.1128/spectrum.02536-22)
Supplement: Supplemental file 1 — Tables S1 and S2 and Fig. S1 and S2. Download spectrum.02536-22-s0001.pdf, PDF file, 0.8 MB [file spectrum.02536-22-s0001.pdf]

Hydroquinones including tetrachlorohydroquinone inhibit *Candida albicans* biofilm formation by repressing hyphae-related genes

Yong-Guy Kim[a], Jin-Hyung Lee[a], Sunyoung Park, Sagar Kiran Khadke, Jae-Jin Shim*, and Jintae Lee*

School of Chemical Engineering, Yeungnam University, Gyeongsan, Republic of Korea

[a]Yong-Guy Kim and Jin-Hyung Lee contributed equally to this article. Author order was determined alphabetically.

*Address correspondence to

Jae-Jin Shim, jjshim@ynu.ac.kr. Phone: +82-53-810-2587

Jintae Lee, jtlee@ynu.ac.kr. Phone: +82-53-810-2533, Fax: +82-53-810-4631

Running Title: Antibiofilm activities of hydroquinones against *Candida*.

Table S1. ADME profile of tetrachlorohydroquinone (TCHQ) and hydroquinone (HQ). The profile is an amalgamation of the ADME properties of TCHQ and HQ from the online webservers, viz. PreADMET, Molinspiration and GUSAR were accessed.

**Property**	**TCHQ**	**HQ**
Lipinski rule of five	Suitable	Suitable
Lipinski rule of five violations	0	0
Plasma protein binding	100%	96%
Blood brain barrier permeability	7.33282	1.56261
Skin permeability	-1.90321	-3.23085
Human intestinal absorption	93.74%	87.42%
Caco2	14.3816	16.5499
Mouse carcinogenicity	Negative	Negative
Rat carcinogenicity	Positive	Positive
Acute algae toxicity	0.008796	0.107092
Acute fish toxicity (medaka)	0.00146411	0.573193
Acute fish toxicity (minnow)	0.00192097	0.174334
In vitro hERG inhibition	Low	Low
miLogP	3.92	0.98
Mol volume	154.22	100.08
TPSA	40.46	40.46
GPCR ligand	-0.83	-3.02
Ion channel modulator	-0.47	-2.48
Kinase inhibitor	-0.91	-3.07
Nuclear receptor ligand	-0.88	-2.84
Protease inhibitor	-1.27	-3.20
Enzyme inhibitor	-0.46	-2.66
Rat IP LD50 classification	Class 4 in AD	Class 4 in AD
Rat IV LD50 classification	Class 4 in AD	Class 4 in AD
Rat oral LD50 classification	Class 5 in AD	Class 4 in AD
Rat SC LD50 classification	Class 4 in AD	Class 4 in AD

Table S2. Primer sequences used for qRT-PCR.

Gene	Function	Primer
ALS1	Cell-surface adhesion, adhesion/invasion, virulence	Forward 5'-AGC TGT TGC CAG TGC TTC-3'<br>Reverse 5'-AAT GTG TTG GTT GAA GGT GAG-3'
ALS3	Cell wall adhesion; epithelial adhesion	Forward 5'-CAA CAT CAA CCA ACC AAT CTC-3'<br>Reverse 5'-TGA ATA ACA GAA CCA GAT CCG-3'
CHK1	Histidine kinase; 2-component signaling, cell wall synthesis	Forward 5'-AGT GAA TTT GGT AGA AAT GGA TGA GA-3'<br>Reverse 5'-CGA AAC CTT CTG GCT GCT TAC-3'
CHT4	Chitinase; similar to S. cerevisiae sporulation-specific Cts2p	Forward 5'-GTA CGA TTG AAT TTG CTG AG-3'<br>Reverse 5'-TTG GAT GAA CTC CCT TGT TA-3'
ECE1	Candidalysin, cytolytic peptide toxin essential for mucosal infection; hypha-specific protein	Forward 5'-CCA GAA ATT GTT GCT CGT GTT GCC A-3'<br>Reverse 5'-TCC AGG ACG CCA TCA AAA ACG TTA G-3'
EFG1	Transcription factor, biofilm formation, hyphal growth, adhesion, virulence	Forward 5'-TAT GCC CCA GCA AAC AAC TG-3’<br>Reverse 5'-TTG TTG TCC TGC TGT CTG TC-3’
ERG3	C-5 sterol desaturase; hyphal growth and virulence	Forward 5'-CAT AAA CCT CAT CAC AAG TGG ATT G-3’<br>Reverse 5'-AAA GAT TGG AAG AACCCATCAACT-3’
ERG11	Sterol 14-demethylase; ergosterol biosynthesis, drug resistance, biofilm	Forward 5'-AAG AAT CCC TGA AAC CAA-3'<br>Reverse 5'-CAG CAG CAG TAT CCC ATC-3'
FKS1	1,3-Beta-D-glucan synthase; cell wall synthesis and maintenance	Forward 5'-CGT GAA ATT GAT CAT GCC TGT AC-3'<br>Reverse 5'-AAC CCT TCT GGG CTC CAA A-3'
HWP1	Hyphal cell wall protein; biofilm	Forward 5'-TTG TTT GCG TCA TCA AGA CTT TG-3'<br>Reverse 5'-GTC TTC ATC AGC AGT AAC ACA ACC A-3'
IFD6	Aldo-keto reductase; biofilm	Forward 5'-TTG GGA AGA TTT TGA TCC TGT TG-3'<br>Reverse 5'-CGA GTG CAT GAT TTC TTC ATA AGT G-3'
RBT5	GPI-linked cell wall protein; biofilm	Forward 5'-CTG CTG AAA GTT CTG CAC CA - 3'<br>Reverse 5'-GCT TCA ACG GAA ACA GAA GC - 3'
TEC1	TEA/ATTS transcription factor	Forward 5'-GGC CAT GAG AGA ACA ATA TA-3'<br>Reverse 5'-GTC TTT CCA TTT CTA AAT CAC-3'
UCF1	Upregulated by cAMP in	Forward 5'-ATG GCG GGA AAG AAA AAG TC-3'

	filamentous growth; biofilm	Reverse 5'-CCC AAG TTT CAT CAC GAA CA-3'
UME6	Zn(II)2Cys6 transcription factor; hyphal extension, virulence, adherence	Forward 5'-AGC ACC AAA TTC GCC TTA TG-3'<br>Reverse 5'-AGG TTG AGC TTG CTG CAG TT-3'
YWP1	Secreted yeast wall protein; biofilm dispersal	Forward 5'-GTT CCA TTT TTC CAA GTT CAT TTA G-3'<br>Reverse 5'-TCA AGA GTA GAA CCT TCA AGA GCA G-3'
ZAP1	Zinc-regulated transcription factor	Forward 5'-CGA CTA CAA ACC ACC AGC TTC ATC-3'<br>Reverse 5'-CCC CTG TTG CTC ATG TTT TGT T-3'
RDN18	18S ribosomal RNA; Housekeeping	Forward 5'-AGA AAC GGC TAC CAC ATC CCA-3'<br>Reverse 5'-CGA ATG GGC CCT GTA TCG T-3'

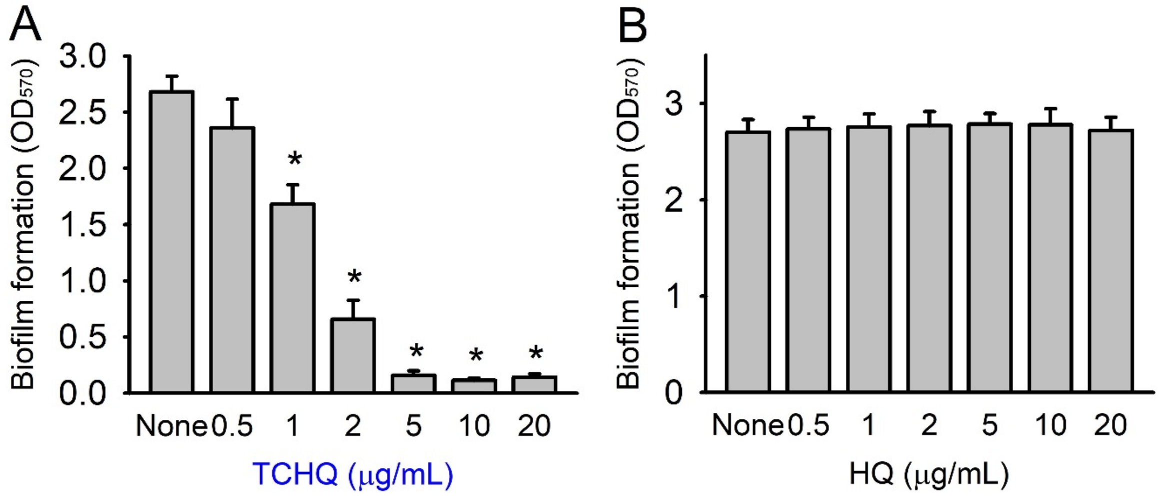

Fig. S1. Inhibitory effect of hydroquinones on biofilm formation by *C. albicans* ATCC 10231. The antibiofilm activity of tetrachlorohydroquinone (TCHQ) and hydroquinone (HQ) on *C. albicans* ATCC 10231 (A and B) were investigated in 96-well polystyrene plates for 24 h. * = $P < 0.05$ *vs.* non-treated controls.

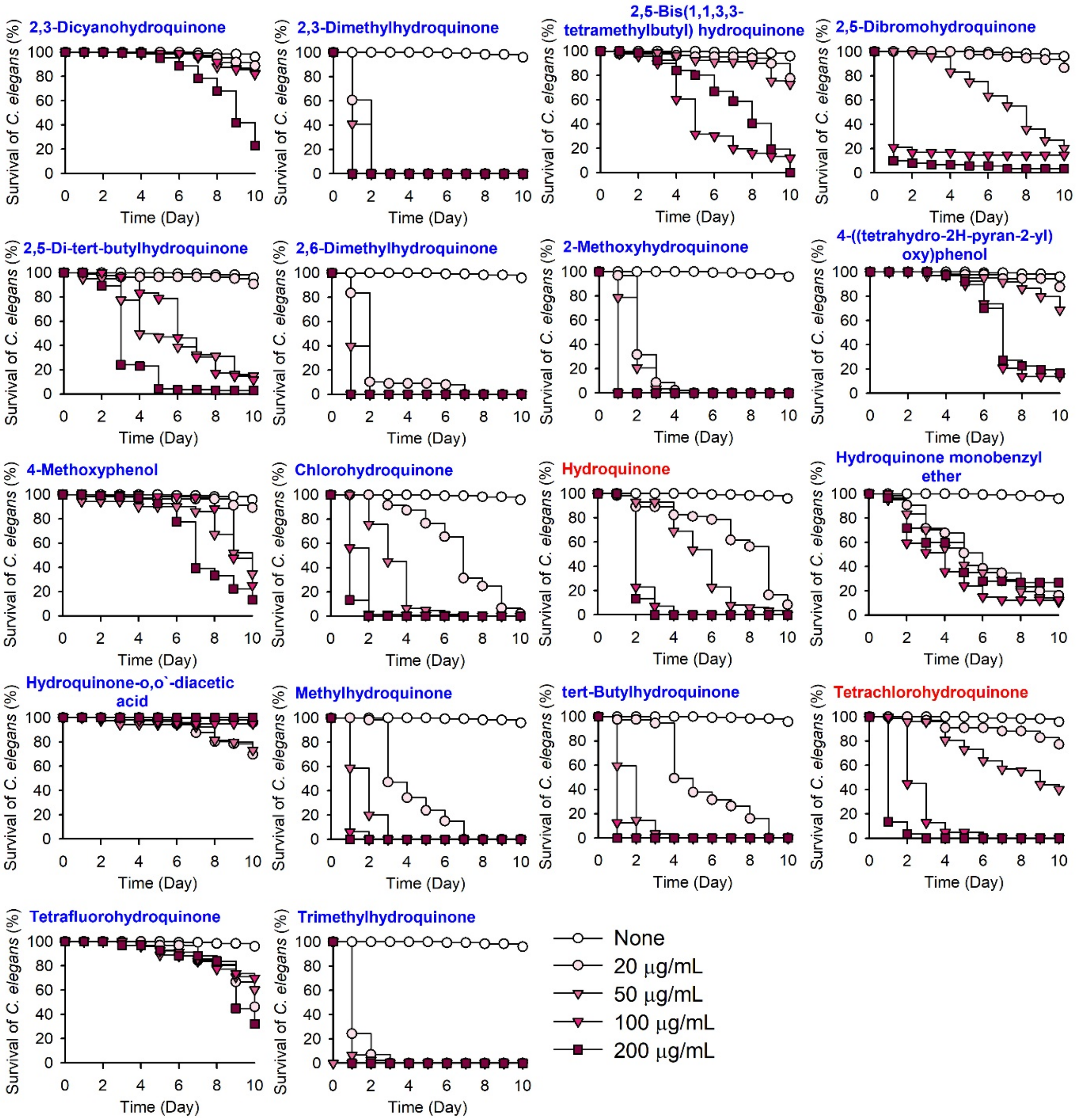

Fig. S2. Toxicities of hydroquinones in the nematode models. *C. elegans* survival was assessed in the presence and absence of hydroquinones (0, 20, 50, 100, and 200 µg/mL) for 10 days.
